# Identification of MET fusions as novel therapeutic targets sensitive to MET inhibitors in lung cancer

**DOI:** 10.1186/s12967-023-03999-7

**Published:** 2023-02-25

**Authors:** Dantong Sun, Weizheng Wu, Li Wang, Jialin Qu, Qiman Han, Huiyun Wang, Shanai Song, Ning Liu, Yongjie Wang, Helei Hou

**Affiliations:** 1 Department of Medical Oncology, National Cancer Center/National Clinical Research Center for Cancer/Cancer Hospital, Chinese Academy of Medical Sciences and Peking Union Medical College, Beijing, 100021 China; 2 State Key Laboratory of Molecular Oncology, National Cancer Center/National Clinical Research Center for Cancer/Cancer Hospital, Chinese Academy of Medical Sciences and Peking Union Medical College, Beijing, 100021 China; 3 Department of General Surgery, Affiliated Hospital of Zunyi Medical University, Zunyi, 563000 Guizhou Province China; 4 Department of Medical Oncology, Shanghai Pulmonary Hospital &, Thoracic Cancer Institute, Tongji University School of Medicine, Shanghai, 200433 China; 5 Department of Radiation Oncology, Shandong Cancer Hospital and Institute, Shandong First Medical University and Shandong Academy of Medical Science, Jinan, 250117 Shandong China; 6 Department of Radiation Oncology, Peking University Third Hospital, Beijing, 100191 China; 7 Department of Oncology, the Affiliated Hospital of Qingdao University, No. 7 Jiaxing Road, Qingdao, 266000 Shandong China; 8 Department of Thoracic Surgery, the Affiliated Hospital of Qingdao University, No. 59 Haier Road, Qingdao, 266000 Shandong China

**Keywords:** Lung cancer, Driver mutations, MET fusions, Tyrosine kinase inhibitors, LAVA database

## Abstract

**Introduction:**

Alterations in the MET gene, including amplifications and exon 14 skipping mutations, have been identified as actionable oncogenic alterations. However, MET fusions are rarely detected in lung cancer, and their sensitivity to therapeutics has not been systematically analyzed.

**Methods:**

The data from 30876 lung cancer patients from the LAVA database and 7966 patients from cBioPortal database were screened. Basic demographic and clinical information for the patients harboring MET fusions were collected. A lung squamous cell cancer patient harboring a novel EML4-MET fusion was treated with crizotinib. Additionally, a literature review was performed to summarize the cases of patients harboring MET fusions and their treatment information.

**Results:**

MET fusions were found in only 0.2% to 0.3% of lung cancer patients and appeared in almost all exons of the MET gene. Intragenic MET fusions were found in 52.6% (41/78) of the included patients. Crizotinib was effective for MET fusions, including a novel identified EML4-MET fusion, even after the failure of multiple lines of treatment. This result suggested that acquired MET fusions become more regionally selective, as they usually occurred in exons encoding the extracellular region. Interestingly, the MET-fused genes in primary MET fusions or acquired MET fusions were very different, which indicated the different functions and influences of the disease.

**Conclusion:**

MET fusions are rare, and half of the fusion types were intragenic fusions. Lung cancer patients harboring primary or acquired MET fusions could benefit from crizotinib. In addition, EML4-MET was first reported in this study as a novel MET fusion type.

**Supplementary Information:**

The online version contains supplementary material available at 10.1186/s12967-023-03999-7.

## Introduction

Lung cancer, for which the 5-year overall survival (OS) is less than 20% in China, ranks first among all malignancies in cancer-related mortality [1] and is a serious public health situation. The management of advanced lung cancer patients has developed rapidly and relies on the wide usage of novel therapeutics, including targeted therapy and immunotherapy. Specifically, patients harboring actionable genomic alterations, such as epidermal growth factor receptor (EGFR), anaplastic lymphoma kinase (ALK) and ROS1 alterations, could benefit from the corresponding targeted therapy and achieve a better outcome with targeted therapy than with conventional treatment [2–4]. With the development of targeted sequencing technology, more putative genomic targets have been revealed and widely used clinically as either prognostic predictors or drug targets for lung cancer patients.

The MET gene is located on the 7th chromosome of the human genome and encodes the MET protein, which serves as the receptor of hepatocyte growth factor (HGF) that participates in the biological regulation of cell proliferation [5]. On the other hand, alterations in the MET gene have been identified as actionable oncogenic alterations [5], and MET gene amplification plays an important role in the growth and survival of non-small cell lung cancer (NSCLC) [6] by enhancing binding to receptor tyrosine kinases (RTKs), which then initiates a series of signal transduction pathways associated with proliferation and metastasis [7]. In addition to MET gene amplifications, mutations in the MET gene, especially exon 14 skipping alterations, are other gain-of-function alterations in MET. In NSCLC patients harboring MET mutations, the administration of selective MET-tyrosine kinase inhibitors (TKIs), such as capmatinib, tepotinib and savolitinib, or other TKIs covering MET mutations, such as crizotinib, has been proven in previous studies to achieve a satisfactory response [8–10]. Immunotherapy is another treatment option for NSCLC patients with MET alterations [11–13]. Recently, a series of reports revealed novel detectable alterations of MET, MET fusions, which are involved in the development of lung cancer and exhibit a special response to the given treatment [14–20]. However, MET fusions are rare in lung cancer, and a systematic analysis of patients harboring this kind of genomic alteration is lacking. In this study, we selected 78 lung cancer patients harboring MET fusions, including 56 patients from the Burning Rock® LAVA database (P.R. China) and 22 patients from the cBioPortal of Cancer Genomics (the cBioPortal) database [21, 22], to elucidate the incidences and types of MET fusions, the responses to treatment, and the concomitant genomic alterations in lung cancer patients harboring primary or acquired MET fusions. In addition, we are the first to report, to the best of our knowledge, the novel EML4-MET fusion in an NSCLC patient who responded dramatically to crizotinib treatment after the failure of multiple lines of anticancer treatments.

## Methods

### Patients’ screening and characteristics collection from the online and real-world databases

We first collected the incidence data from a pan-cancer study, MSK MetTropism (MSK, Cell 2021), of the cBioPortal database to analyze the incidences of MET gene alterations. No special pre-selection for patients in this dataset, such as tumor types, age, sex and race, in order to fully understood the incidences of MET fusions and the distribution of MET fusions among different cancer types. At the same time, we particularly screened lung cancer patients in the cBioPortal database, with a sample size of 7966 patients. As listed in Table 1, the demographic and basic clinical data for the selected patients harboring MET fusions were collected, including age, sex, tumor stage, smoking history, pathological types and MET-fusion types. In addition, previous treatment information, including information on treatment with EGFR and ALK tyrosine kinase inhibitors (TKIs), was collected, and information on the concomitant genomic alterations of the patients from the LAVA database was acquired.

**Table 1:** Basic characteristics of enrolled lung cancer patients harboring MET fusions

Characteristics	The cBioPortal (n = 22)	LAVA database (n = 56)
Age (average age, range)	69 (49–83)	59 (27–83)
Sex		
Male	7	31
Female	15	25
Tumor stage		
I–III	5	14
IV	7	42
NA	10	0
Smoking history		
Ever	13	0
Never	1	0
NA	8	56
Pathological types		
Adenocarcinoma	22	50
Squamous cell carcinoma	0	3
Small cell lung cancer	0	1
Other types	0	2
MET fusions types		
Intragenic fusions	14	27
Other types	8	29

*NA* not applicable

Then, as of August 15th 2022, a total of 30,876 lung cancer patients from the Burning Rock® LAVA database (Burning Rock Biotech, Guangzhou, China) were screened via the system. The search term used was “lung cancer”, and no other pre-selection for the lung cancer patients. The LAVA database (system) is a targeted sequencing data cloud platform built by Burning Rock® Biotech, which can quickly query patients in the database through some simple functions, and can also see the overall data analysis of all patients sent by doctors, besides, the data is anonymous and protects patients’ privacy. Each piece of information in the database is real-world data, formed by Burning Rock® via collecting basic patients’ information, obtaining patients’ tissue sections for targeted sequencing, and uploading it to the database. The database contains patients from various hospitals, whose pathological sections were sent by clinicians, and the basic information of patients will be collected before genetic testing, such as: sex, age, pathological type and disease stage, etc., and genetic testing will be carried out only after all information is collected, and therefore no missing data in this database as well as in the process of database search in this study. All patients included in the database gave informed consent.

### DNA isolation and capture-based targeted DNA sequencing

DNA isolation and targeted sequencing were performed in Burning Rock® Biotech, a commercial clinical laboratory accredited by the College of American Pathologist (CAP) and certified by the Clinical Laboratory Improvement Amendments (CLIA), according to optimized protocols as described previously [23, 24]. Briefly, tissue DNA was extracted from formalin-fixed, paraffin-embedded (FFPE) tumor tissues using QIAamp DNA FFPE tissue kit (Qiagen, Hilden, Germany) and circulating cell-free DNA (cfDNA) was extracted from 4–5 ml of liquid samples using a QIAamp Circulating Nucleic Acid kit, according to the manufacturer’s standard protocol (Qiagen, Hilden, Germany). Fragments between 200 and 400 bp from the sheared tissue DNA and cfDNA were purified (Agencourt AMPure XP Kit, Beckman Coulter, CA, USA), hybridized with capture probes baits, selected with magnetic beads, and amplified. Target capture was performed using a commercial panel consisting of 168 lung cancer-related genes (Burning Rock® Biotech Ltd., Guanzhou, China), spanning 0.273 megabases (Mb) of the human genome. The quality and the size of the fragments were assessed by high sensitivity DNA kit using Bioanalyzer 2100 (Agilent Technologies, CA, USA). Indexed samples were sequenced on Nextseq 500 (Illumina, Inc., CA, USA) with paired-end reads and average sequencing depth of 1,000 × for tissue samples and 10,000 × for liquid biopsy samples.

### Sequence data analysis

Sequence data were mapped to the reference human genome (hg19) using Burrows-Wheeler Aligner version 0.7.10 [25]. Local alignment optimization, duplication marking and variant calling were performed using Genome Analysis Tool Kit version 3.2 [26], and VarScan version 2.4.3 [27]. Tissue and plasma samples were compared against their own white blood cell control to identify somatic variants. Variants were filtered using the VarScan fpfilter pipeline, loci with depth less than 100 were filtered out. Base calling in plasma and tissue samples required at least 8 supporting reads for single nucleotide variations (SNVs) and 2 and 5 supporting reads for insertion-deletion variations (Indels), respectively. Variants with population frequency over 0.1% in the ExAC, 1000 Genomes, dbSNP or ESP6500SI-V2 databases were grouped as single nucleotide polymorphisms (SNPs) and excluded from further analysis. Remaining variants were annotated with ANNOVAR (2016–02-01 release) [28] and SnpEff version 3.6 [29]. Analysis of structural variations (SVs) was performed using Factera version 1.4.3 [30]. Copy number variations (CNVs) were analyzed based on the depth of coverage data of capture intervals. Coverage data were corrected against sequencing bias resulting from GC content and probe design. The average coverage of all captured regions was used to normalize the coverage of different samples to comparable scales. Copy number was calculated based on the ratio between the depth of coverage in tumor samples and average coverage of an adequate number (n > 50) of samples without CNVs as references per capture interval. CNV is called if the coverage data of the gene region was quantitatively and statistically significant from its reference control. The limit of detection for CNVs is 1.5 for copy number deletion and 2.64 for copy number amplifications. The MSI status of tumor and plasma samples were determined based on a read-count-distribution-based method as previously published [31, 32].

### Literature review and case report

In this study, we collected the published cases of lung cancer patients harboring MET fusions. Basic information, including publication time, the types and appearance times of MET fusions, tumor stage, concomitant EGFR/ALK mutations, pathological types and treatment information, was collected, as displayed in Table 2. Specifically, we report here in this study a novel MET fusion type, EML4-MET, which had not been reported in the published cases to the best of our knowledge, and a dramatic response to crizotinib in a previously treated lung squamous cell carcinoma (LUSC) patient was observed.

**Table 2:** Reported lung cancer patients harboring MET fusions and the corresponding treatment information

Reported time [Ref.]	MET fusion types	Primary/ acquired	Stage	EGFR/ALK mutations	Pathological types	The PFS of crizotinib treatment and best overall response
2017 [14]	KIF5B-MET	Primary	IV	WT	LUAD	10.0 months, PR
2017[18]	HLA-DRB1-MET	Primary	IV	WT	LUAD	8.0 months, complete resolution of lung nodules
2018 [18]	KIF5B-MET	Primary	IV	WT	LUAD	8.0 months, PR
2018 [18]	STARD3NL-MET	Primary	IV	WT	LUAD	14.0 months, PR
2018 [18]	MET-ATXN7L1	Primary	IV	WT	LUAD	4 months, PR
2018[18]	MET-UBE2H	Acquired	IV	EGFR exon 19 deletion	LUAD	6.5 months, PR
2019 [15]	CAV1-MET	Acquired	IV	EGFR exon 21 L858R	LUAD, SCLC transformation	NR (plus osimertinib), PR
2020 [19]	HLA-DRB1-MET	Primary	IV	WT	LUAD	7.0 months, complete resolution of lung/brain nodules
2021[18]	HLA-DRB1-MET	Primary	IV	WT	LUAD	NR, CR
2022 [16]	ARL1-MET	Primary	IV	WT	LUAD	5.0 months, PR
2022 [17]	CUX1-MET	Acquired	IV	EGFR exon 18 G719D and exon 21 L861Q	LUAD	9.0 months(plus icotinib), PR
2022 [20]	MET-DSTN	Acquired	IV	EGFR exon 21 L858R	LUAD	NR (plus gefitinib), CR

*LUAD* lung adenocarcinoma, *SCLC* small cell lung cancer, *WT* wild type, *NR* not reached, *PR* partial response, *CR* complete response, *ICC* intrahepatic cholangiocarcinoma

### Statistical analysis

The fusion sites of the MET gene in patients from the LAVA database were detected, and the fusions in MET gene exons were then matched via the cBioPortal database for functional detection purposes. The locations of genes that fused with the MET gene were acquired from The Human Protein Atlas (https://www.proteinatlas.org/) database. The “GenVisR” package was used to generate the Oncoprint waterfall plots and the data were analyzed using the statistical software R, version 3.6.2. The Metascape [33] online tool was used for functional enrichment in this study.

## Results

### Relative data for MET fusions in lung cancer patients from the cBioPortal and LAVA databases

**Fig. 1:**
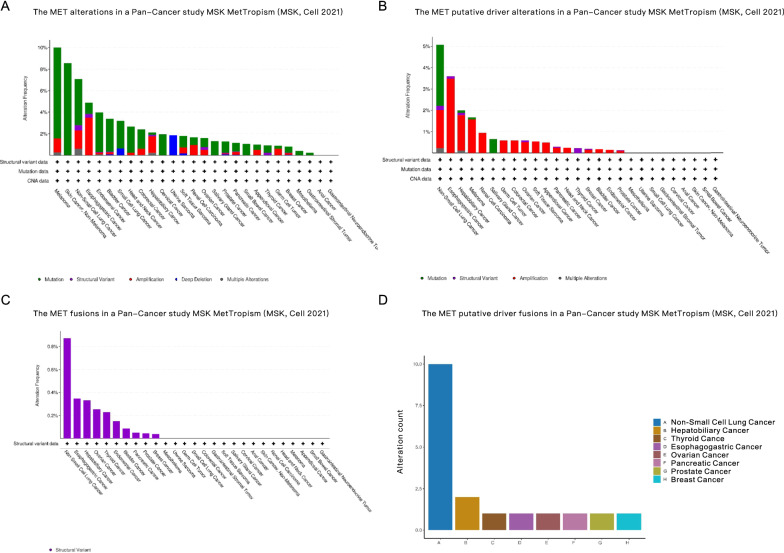
MET variations in the pan-cancer study, MSK MetTropism (MSK, Cell 2021), of the cBioPortal database. **A** MET variations in the pan-cancer study. **B** MET putative driver variations in the pan-cancer study. **C** MET fusions in the pan-cancer study. D. MET putative driver fusions in the pan-cancer study

The selection of patients from relevant databases and the purposes for the analysis were diaplayed in Additional file 1: Figure S1. In the pan-cancer dataset, MET alterations were common, as shown in Fig. 1A, especially (over 5%) in melanoma, skin cancer (nonmelanoma) and NSCLC. Not all alterations of the MET gene are considered driver mutations in cancers. As displayed in Fig. 1B, over 5% of NSCLC patients harbored putative driver mutations of MET, including MET mutations, MET amplifications, MET structural variants and other undefined mutations. Especially for MET fusions in cancers, as shown in Fig. 1C, although it was rare among all types of cancers, NSCLC patients had a significantly higher incidence of MET fusions as well as the types of MET fusions, which are considered putative driver mutations (Fig. 1D).

**Fig. 2:**
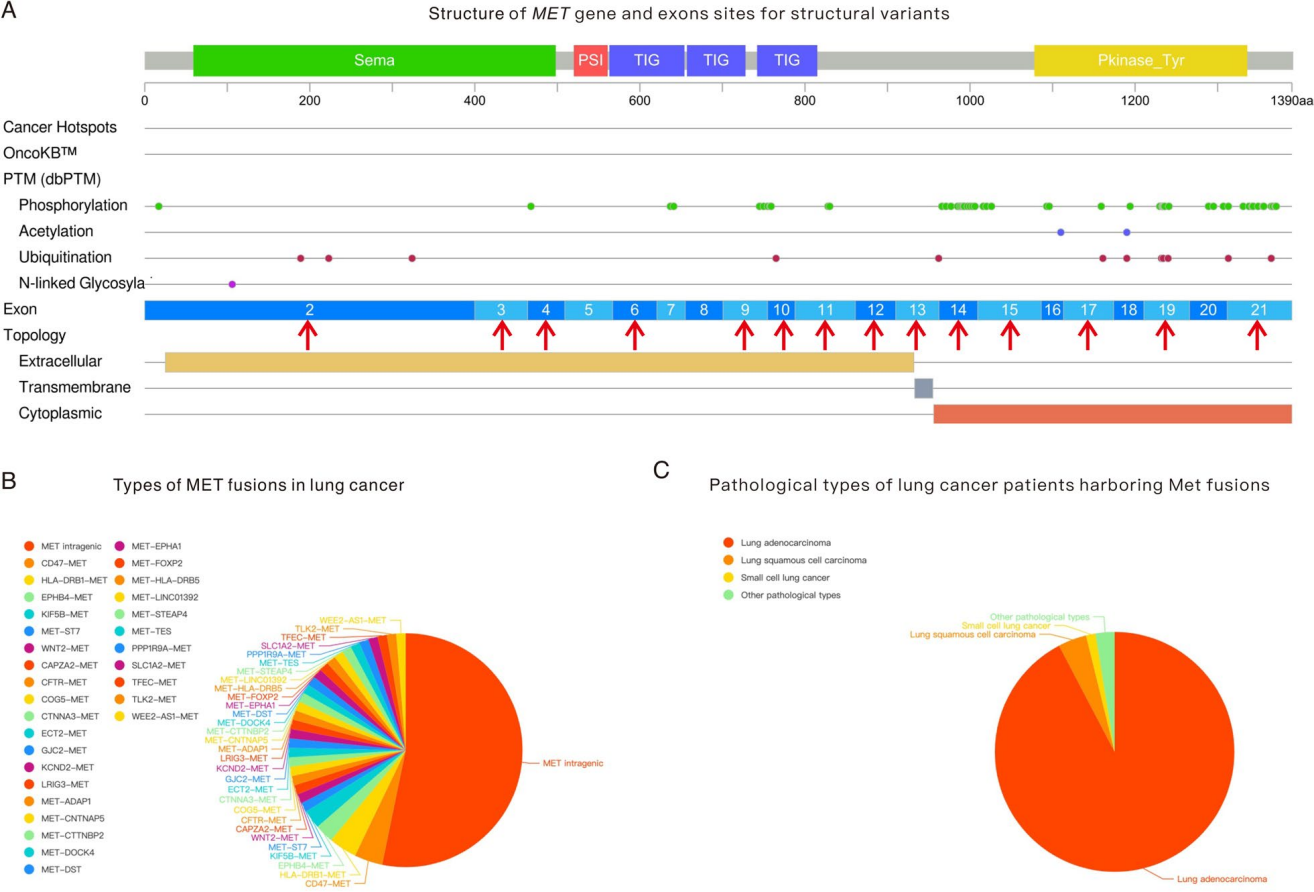
Fusion sites and types of MET fusions in selected lung cancer patients. **A** MET gene structure and exons that participate in the fusion. **B** Types of MET fusions in lung cancer patients. **C** Pathological types of patients harboring MET fusions

In the two databases involved in this study, MET fusions were rare in lung cancer patients, being found in 0.2% (56/30876, LAVA database) to 0.3% (22/7966, the cBioPortal database) of all patients. From the point of view of MET gene structure, as shown in Fig. 2A, MET fusions appeared in almost all of the exons of MET genes according to the targeted sequencing data of the selected patients, including exon 2, exon 3, exon 4, exon 6, exon 9, exon 10, exon 11, exon 12, exon 13, exon 14, exon 15, exon 17, exon 19 and exon 21. These exons were responsible for the encoding of different regions of the MET protein, including the extracellular region, transmembrane region and cytoplasmic region. Regarding the fusion types of the MET gene, intragenic fusions of MET were found in 52.6% (41/78) of involved patients (cBioPortal: 14/22, 63.6%; LAVA database: 27/56, 48.2%), as shown in Table 1. Despite intragenic fusions of MET, a series of genes were found to participate in fusion with the MET gene, as displayed in Fig. 2B, including CD47 (3.8%), HLA-DRB1 (3.8%), EPHB4 (2.6%), KIF5B (2.6%), ST7 (2.6%), WNT2 (2.6%), CAPZA2 (1.3%), CFTR (1.3%), COG5 (1.3%), CTNNA3 (1.3%), ECT2 (1.3%), GJC2 (1.3%), KCND2 (1.3%), LRIG3 (1.3%), ADAP1 (1.3%), CNTNAP5 (1.3%), CTTNBP2 (1.3%), DOCK4 (1.3%), DST (1.3%), EPHA1 (1.3%), FOXP2 (1.3%), HLA-DRB5 (1.3%), LINC01392 (1.3%), STEAP4 (1.3%), TES (1.3%), PPP1R9A (1.3%), SLC1A2 (1.3%), TFEC (1.3%), TLK2 (1.3%), and WEE2-AS1 (1.3%). Most of the genes are located on chromosome 7 (55.8%), while other genes are located on chromosomes 1, 2, 3, 4, 5, 6, 8, 9, 10, 12, 13, 17, 18 and 19. In addition, 35.7% (20/56) of patients in the LAVA database harbored concomitant MET fusions and MET amplifications, only one patient harbored concomitant MET fusions and MET Mutation (MET exon 14 skipping).

To elucidate the characteristics of lung cancer patients harboring MET fusions, we summarized the basic demographic and clinical information of the 78 selected patients in Table 1. MET fusions more frequently appeared in lung adenocarcinoma (LUAD) patients (72/78, 92.3%) than in patients with other pathological types of lung cancer, as shown in Fig. 2C. All 22 patients from the cBioPortal database were LUAD patients, while 89.3% (50/56) of patients in the LAVA database were diagnosed with LUAD. The others were diagnosed with LUSC (3/56, 5.4%), small cell lung cancer (SCLC, 1/56, 1.8%) and other pathological types (2/56, 3.5%). The average age of the 22 patients in the cBioPortal database was 69 years, ranging from 49 to 83 years, while that of the patients in the LAVA database was 59 years, ranging from 27 to 83 years. No significant difference in sex was found in the 78 patients (males: 38; females: 40). 59.1% patients from the cBioPortal (13/22) harboring MET fusions were smoker or ever, but no relevant data was available of patients from LAVA database.

### The treatment for lung cancer patients harboring MET fusions: From published cases to a unique LUSC patient harboring a novel EML4-MET fusion

As described in the results above, MET fusions are rare in lung cancer patients. To determine what kind of therapeutics would benefit lung cancer patients harboring MET fusions, we performed a literature review of cases published by September 2022. As summarized in Table 2, a total of 12 patients harboring primary or acquired MET fusions with detailed treatment information available were included. All patients were diagnosed with stage IV LUAD. For the 8 patients harboring primary MET fusions, the progression-free survival (PFS) with crizotinib treatment ranged from 4.0 months to 14.0 months. Surprisingly, in the best overall response for the 8 patients harboring primary MET fusions, 5 patients achieved a partial response (PR), and the complete resolution of cancerous nodules was detected in 3 patients. Another 4 patients were found to harbor baseline EGFR-sensitive mutations, as listed in Table 2. After the failure of EGFR-TKI treatment, all of the patients harbored acquired MET fusions and received the regimen of crizotinib plus EGFR-TKIs for their subsequent treatment. The efficacy of crizotinib combined therapy was still robust in this group of patients. Two patients reached the PFS endpoint, with a PFS of 6.5 months and 9.0 months, respectively. The other 2 patients did not reach the PFS endpoint. Regarding the best overall response, 3 patients achieved a PR after combined therapy, and 1 patient achieved a complete response (CR). According to the outcomes of these cases, single-agent crizotinib or crizotinib combination therapy can serve as a suitable treatment option for patients harboring primary and acquired MET fusions.

**Fig. 3:**
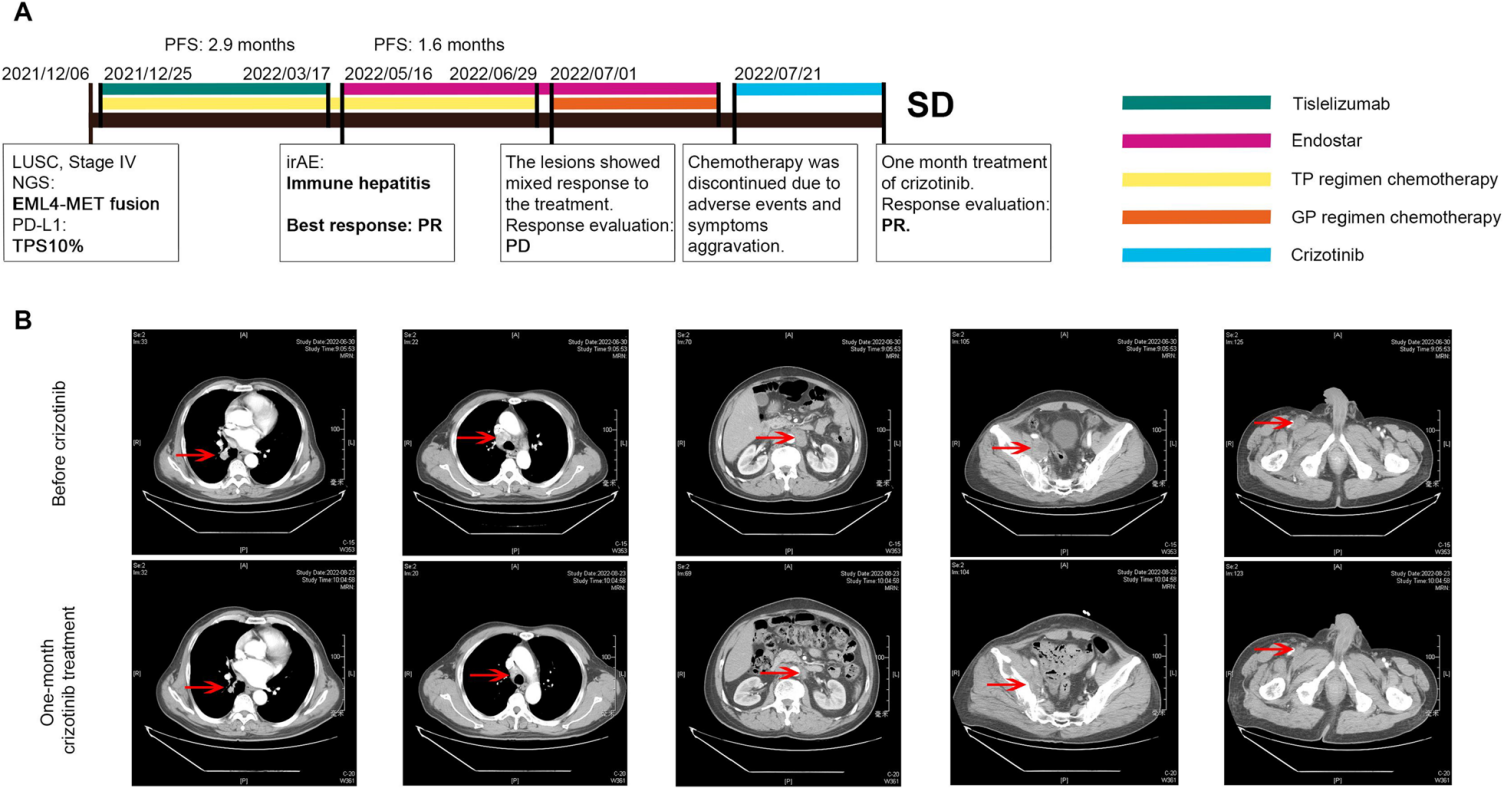
A novel EML4-MET fusion in a patient who responded to crizotinib treatment. **A** The treatment timeline of the reported patient harboring the EML4-MET fusion. **B** CT scan revealed a significant response to crizotinib in the patient harboring the EML4-MET fusion

The case that we reported here is that of a unique LUSC patient harboring primary EML4-MET. The novel EML4-MET fusion type was not found in the 78 selected patients, nor was it reported in the published cases. The treatment timeline for the patient is displayed in Fig. 3A. The patient was diagnosed with stage IV LUSC on December 6th, 2021, at the Affiliated Hospital of Qingdao University. Next-generation sequencing (NGS) revealed the primary EML4-MET fusion without other common driver mutations. The patient was then administered first-line tislelizumab combined with TP chemotherapy according to the treatment guidelines and PD-L1 expression (TPS: 10%). Unfortunately, the immunotherapy had to be stopped shortly after initiation as a result of immune hepatitis, and the PFS was 2.9 months, while the best overall response was a PR. The patient then received Endostar and TP regimen chemotherapy as the second-line treatment. However, after 1.6 months of treatment, the lesions demonstrated a mixed response to the treatment, and the chemotherapy regimen was changed to the GP regimen after evaluation. However, adverse events (AEs) and aggravating symptoms led to treatment discontinuation. Next, crizotinib was used for the fourth-line treatment of this patient. Interestingly, 1 month of crizotinib treatment showed satisfactory efficacy in this patient. The patient’s symptoms, especially shortness of breath, were alleviated significantly, and the CT scan demonstrated a PR of the systematic lesions, as shown in Fig. 3B. Hence, crizotinib was effective for this MET fusion type, even after the failure of multiple lines of treatment. In addition, the safety of crizotinib treatment for MET fusion patients was tolerable according to the published cases above.

### The analysis of primary MET fusions or acquired MET fusions after the development of EGFR/ALK-TKI resistance in lung cancer patients from the LAVA database

**Fig. 4:**
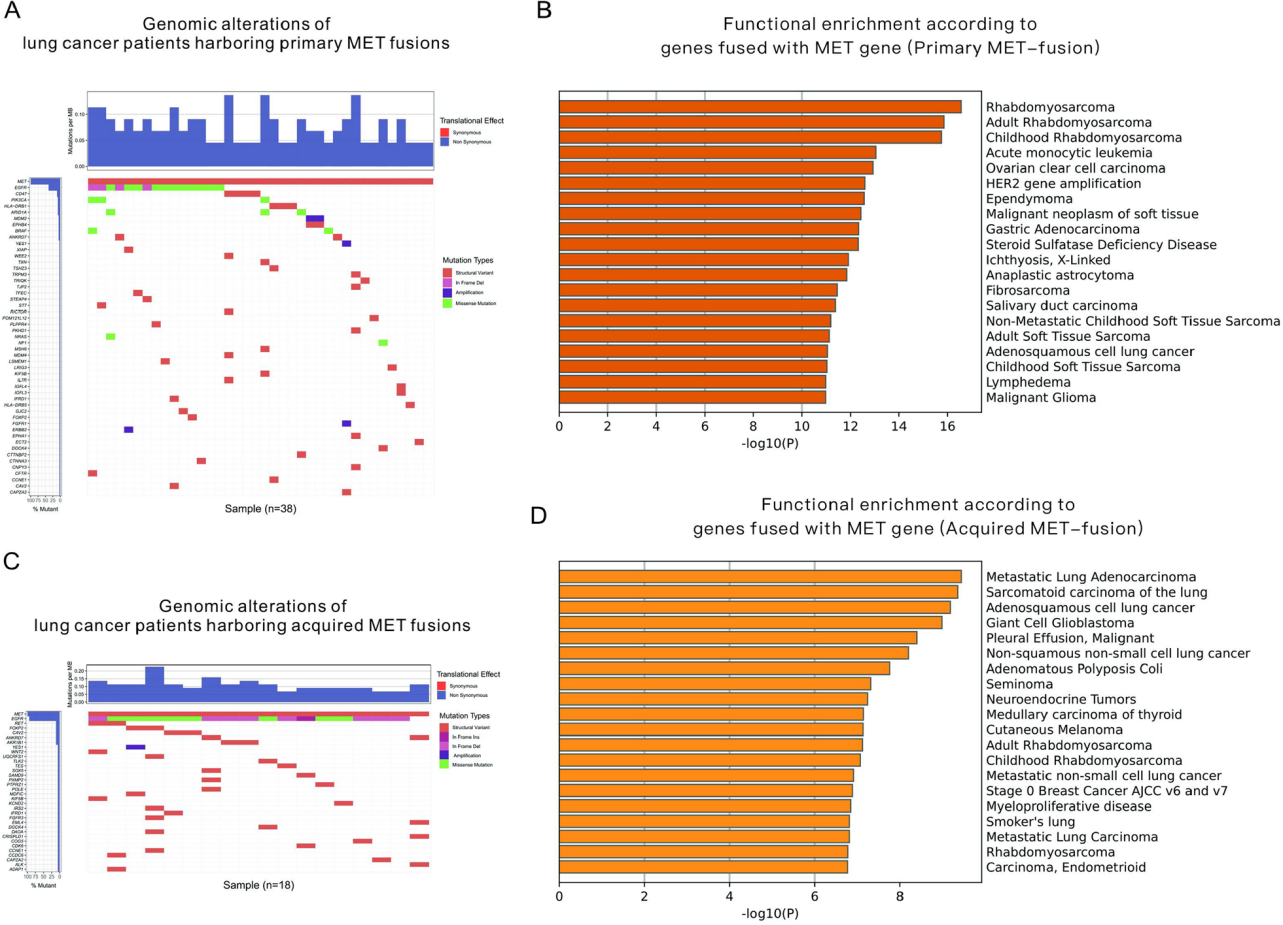
Comparison between primary and acquired MET fusions in lung cancer patients. **A**, **C** Waterfall plots for patients harboring primary and acquired MET fusions. **B**, **D** Functional enrichment based on MET-fused genes in lung cancer patients harboring primary and acquired MET fusions

In 56 selected lung cancer patients from the LAVA database harboring MET fusions, 38 patients had primary MET fusions and 18 patients acquired MET fusions after developing resistance to EGFR/ALK-TKIs, as shown in Table 3. The fusion sites of the MET gene are also shown in Table 3. It is likely that the acquired MET-fused genes usually fused with the MET exons mostly encoding the extracellular region, while the primary MET-fused genes showed no significant difference in MET exon selection. For 38 patients harboring primary MET fusions, the concomitant genomic alterations of these patients are displayed in Fig. 4A, and the functional enrichment is shown in Fig. 4B. For the 22 patients harboring acquired MET fusions, the concomitant genomic alterations of these patients are displayed in Fig. 4C, and the functional enrichment is shown in Fig. 4D. The results demonstrated that the 2 groups of patients had significantly different MET-fused genes. Functional enrichment also revealed differences in function between the MET-fused genes in these 2 groups.

**Table 3:** Comparison of MET fusion sites of enrolled lung cancer patients according to the status of EGFR/ALK mutations

Fusion sites of MET	Primary MET fusions (n = 38)		Acquired MET fusions (n = 18)	
	Number	Location	Number	Location
Introns	25	NA	10	NA
Exons	13		6	
Exon2	1	Extracellular	/	/
Exon3	/	/	2	Extracellular
Exon4	/	/	1	Extracellular
Exon6	/	/	1	Extracellular
Exon10	1	Extracellular	/	/
Exon11	1	Extracellular	1	Extracellular
Exon12	2	Extracellular	/	/
Exon13	1	Transmembrane	/	/
Exon14	2	Cytoplasmic	/	/
Exon15	1	Cytoplasmic	/	/
Exon17			1	Cytoplasmic
Exon19	2	Cytoplasmic	/	/
Exon21	2	Cytoplasmic	/	/
NA	/	/	2	/

*NA* not applicable

## Discussion

The rapid development of targeted sequencing has provided more opportunities for the treatment of lung cancer patients. Accordingly, patients harboring actionable genomic alterations respond differently to therapeutics. MET alterations, including MET mutations, amplifications and gene fusions, are relatively rare genomic alterations in NSCLC patients [34]. Selective MET-TKIs, such as capmatinib, tepotinib, savolitinib, other TKIs covering MET mutations, including crizotinib, serve as efficient therapeutics for patients harboring MET mutations [8–10], but their use in patients with MET amplification and fusions still needs to be evaluated. In the latest research [35], researchers disclosed that MET amplifications can serve as a biomarker for the poor prognosis of patients with NSCLC, while patients harboring MET amplifications respond poorly to immunotherapy. Crizotinib or other MET-TKIs are likely to be the optimal choices for beneficial treatment efficacy. In addition, chemotherapy plus bevacizumab may benefit patients harboring sensitive EGFR mutations and acquired MET amplifications after the failure of EGFR-TKI treatment. In this study, we collected the published cases [14–20] of patients harboring MET fusions, and the findings cast new light on the role of crizotinib in benefiting these patients. The findings suggested that crizotinib as a single agent can serve as a suitable treatment option for patients harboring primary MET fusions, and combined therapy with crizotinib and EGFR-TKIs could significantly benefit patients with EGFR mutations also harboring acquired MET fusions after the development of resistance to EGFR-TKIs. The outcome of the LUSC patient reported in this study also supported the efficacy of crizotinib. Generally, patients harboring MET fusions showed a satisfactory response to crizotinib treatment.

MET fusions are rare in lung cancer patients, which has led to a lack of a systematic understanding of this kind of genomic alteration, both in terms of the genomic appearance and the treatment of patients. To the best of our knowledge, our study is the first to analyze the basic characteristics of lung cancer patients harboring MET fusions. Our analysis showed that MET fusions frequently appeared in LUAD patients, similar to other actionable genomic alterations in lung cancer [2–4]. MET intragenic fusions accounted for most MET fusions in the patients in this study, while a series of genes participated in the fusion in other types of MET fusions in lung cancer. The MET-fused genes were located on different chromosomes of the human genome but mainly on chromosome 7, where the MET gene is located. Therefore, the fusions usually took place within the same chromosome.

Multiple concomitant genomic alterations were detected in lung cancer patients harboring MET fusions, but concomitant MET amplifications (35.7%) seemed to be the most common genomic alteration. There was no selectivity for the fusion sites for the MET gene, and nearly all of the exons could serve as the fusion sites, as well as introns. However, there were differences when comparing the fusion sites between primary MET fusions and acquired MET fusions in treatment-naive patients and EGFR/ALK-TKI-resistant patients. This result suggested that acquired MET fusions become more selective in fusion sites, which usually were in the extracellular region of MET. However, the underlying mechanism and the influence of the fusions on the function of MET remain unclear and need to be further explored. Interestingly, the MET-fused genes in primary MET fusions or acquired MET fusions were very different, which indicated the different functions and influences of the disease. Admittedly, the current study is not without limitations for confirming the role of MET fusions in the development of lung cancer resistance to EGFR/ALK-TKIs, and further exploration of these rare actionable genomic alterations is needed. However, our study first determined the basic characteristics of lung cancer patients harboring primary or acquired MET fusions, and the findings cast new light on their role in targeted therapy.

## Conclusion

MET fusions were rare in lung cancer and appeared in approximately 0.2%-0.3% of patients, most of whom had LUAD. Lung cancer patients harboring MET fusions could benefit from crizotinib treatment. In addition, EML4-MET was first reported in this study as a novel MET fusion type.

## Supplementary Information

**Additional file 1: Figure S1.** The framework for patients’ selection and study purposes.

## Data Availability

All data and material from this study are available.

## Abbreviations

OS: Overall survival
EGFR: Epidermal growth factor receptor
ALK: Anaplastic lymphoma kinase
HGF: Hepatocyte growth factor
RTKs: Receptor tyrosine kinases
NSCLC: Non-small cell lung cancer
TKIs: Tyrosine kinase inhibitors
LUSC: Lung squamous cell carcinoma
LUAD: Lung adenocarcinoma
NGS: Next-generation sequencing
AE: Adverse event
