# Supplementary figures and images for: Identification of MET fusions as novel therapeutic targets sensitive to MET inhibitors in lung cancer
Source: J Transl Med. 2023 Feb 25;21:150. doi: 10.1186/s12967-023-03999-7 (PMC9960416; doi:10.1186/s12967-023-03999-7)

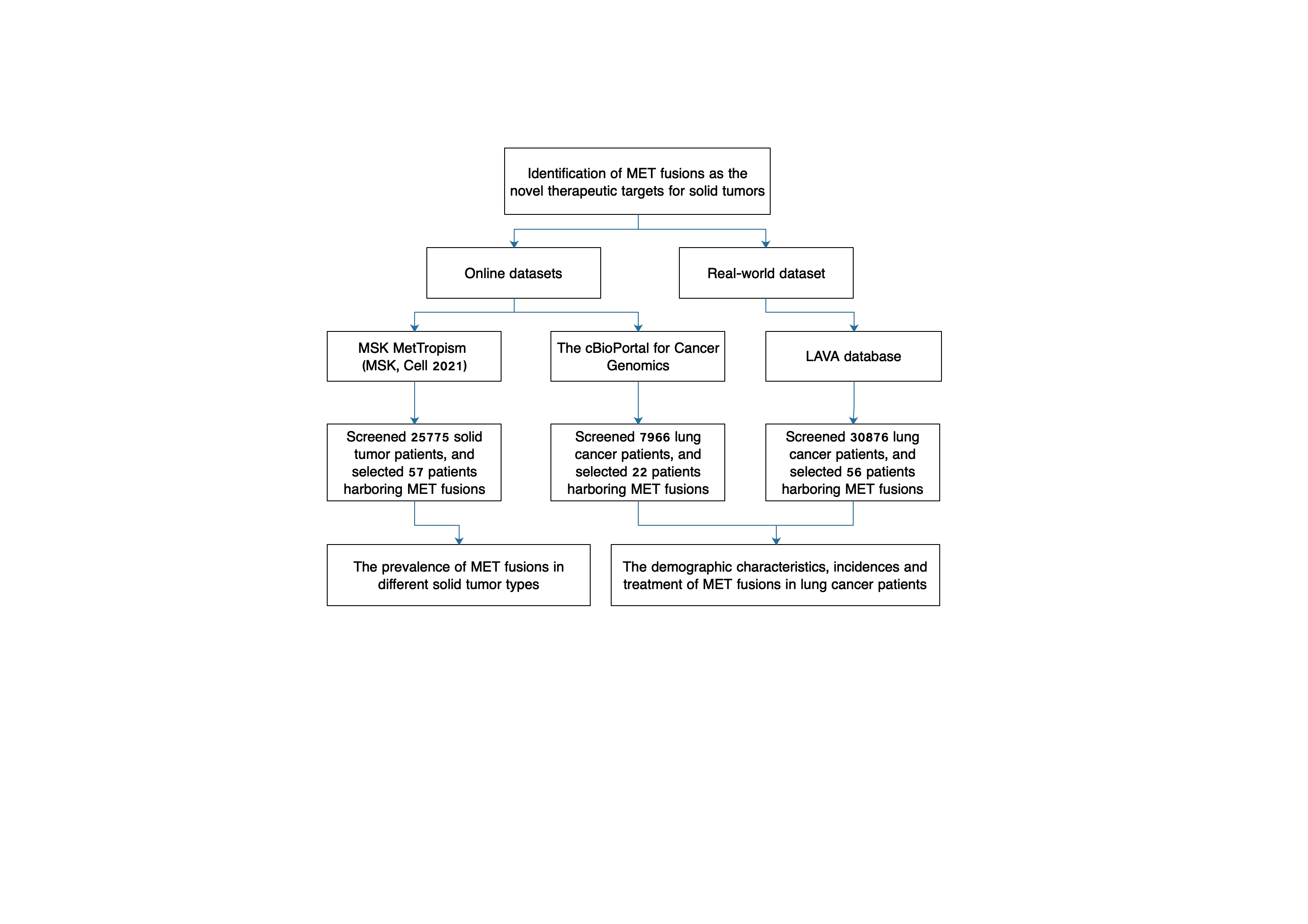

Supplement: Supplementary file 1 — Additional file 1: Figure S1. The framework for patients’ selection and study purposes. [file 12967_2023_3999_MOESM1_ESM.jpg]
